# Epidemiological analysis of syphilis surveillance among entry-exit population at Shanghai Port, China from 2014 to 2022

**DOI:** 10.1186/s13690-023-01176-2

**Published:** 2023-08-24

**Authors:** Quan Jin, Jing Zhang, Jing Xia, Jia Qin, Xuan Zhou

**Affiliations:** Shanghai International Travel Healthcare Center, 15 Jinbang Road, Shanghai, 200032 China

**Keywords:** Syphilis, Port of entry, Entry and exit, Epidemiology

## Abstract

**Objective:**

To investigate the epidemiological characteristics of syphilis cases detected among entry-exit personnel at Shanghai ports from 2014 to 2022 and the changing trend of the syphilis epidemic in the region so as to provide data support for the scientific and effective prevention and control of syphilis at ports.

**Methods:**

From January 2014 to December 2022, the subjects of syphilis screening at Shanghai port were selected. Physical examination and serological testing were used to confirm syphilis. All the data used were downloaded from the HIS system of Shanghai International Travel Healthcare Center. Descriptive epidemiology was used to analyze the characteristics of the detected cases, and the linear trend Chi-square test was used to analyze the trend between groups.

**Results:**

From 2014 to 2022, a total of 918 cases of syphilis were detected among entry-exit personnel at Shanghai port, with a total detection rate of 154.68/100 000. The detection rate was the highest in 2015 and the lowest in 2022, showing a downward trend year by year since 2015. 54.36% of syphilis patients from East Asia were detected. Syphilis cases were reported in all age groups; most cases were under 39 years old, accounting for 36.06%. The syphilis detection rate in males was significantly higher than in females (79.63% vs. 20.4%). The main way of transmission was sexual transmission, accounting for 60.89%, among which male-to-male transmission was the primary way (22.36%).

**Conclusion:**

The detection rate of syphilis among entry-exit personnel at Shanghai port has been decreasing continuously in recent years. Targeted health intervention should be carried out according to the monitoring results.

**Table:** 

**Text box 1. Contributions to the literature**
• The study provides an analysis of the prevalence and epidemiological characteristics of syphilis in the entry-exit population at Shanghai port.
• It highlights that sexual transmission is the primary route of syphilis transmission among entry-exit personnel, and the prevalence of syphilis varies significantly among different regions, with the highest detection rate in East Asia.
• The study reveals a declining trend in syphilis detection rate year by year from 2015 to 2022, suggesting that efforts to combat syphilis in the region have been relatively effective.
• It shows that the detection rate of syphilis was higher in males than in females.

Syphilis is a sexually transmitted infectious disease caused by the Treponema pallidum subspecies. The majority of new cases are caused by sexual transmission, while a smaller portion occurs through vertical transmission [1]. Between 2000 and 2020, the global pooled prevalence was 7·5% (95% CI 7·0–8·0%). Regional variations were observed, with prevalence ranging from 1·9% (1·0–3·1%) in Australia and New Zealand to 10·6% (8·5–12·9%) in Latin America and the Caribbean [2]. The median incidence of syphilis was 17.2 cases per 100,000 in women and 17.7 cases per 100, 000 in men globally. The regions with the highest incidence rates were the Western Pacific (93.0 cases per 100,000), Africa (46.6 cases per 100,000), and America (34.1 cases per 100,000) [3]. In the United States, there was a significant increase in syphilis prevalence and incidence rates, rising by 164% and 175%, respectively, from 2008 to 2018. In 2018, there were 146,000 new syphilis cases in people aged 14–49 years in the United States, with males accounting for 80% of these cases. The risk of syphilis infection was relatively higher in individuals aged 25–49 years [3]. Notably, the global prevalence of syphilis in men who have sex with men (MSM) was found to be 15 times higher than the latest estimate for males in the general population (7.5% vs. 0.5%). Latin America and the Caribbean had the highest incidence of syphilis (10.6% (8.5–12.9%), while Australia and New Zealand had the lowest incidence (1.9% (1.0-3.1%)). Regarding trends over time, the global incidence of syphilis in MSM was estimated to be 7.5% between 2000 and 2020. Studies conducted from 2000 to 2009 and from 2010 to 2020 reported estimated global incidences of 8.9% and 6.6%, respectively. Moreover, the aggregated prevalence rate in East Asia from 2010 to 2020 was lower than that from 2000 to 2009 [2]. Due to a large number of cases, severe disease, wide coverage of the population, and large regional differences, syphilis has become one of the important public health and social problems in the world [4, 5]. At the same time, syphilis is one of the key infectious diseases monitored by the inspection and quarantine institutions of all countries in the world.

According to the statistics released by the Shanghai Municipal Bureau of Statistics, the number of overseas tourists entering Shanghai port and the number of overseas tourists entering Shanghai from other ports of China experienced steady growth from 2014 to 2019, increasing from 7.913 million to 8.972 million each year. However, the outbreak of COVID-19 in 2020 had a significant impact on international travel, leading to a decrease in the cumulative entry of Shanghai port to 1.2862 million in that year. This represents an 85.7% decline compared to the previous year. The numbers gradually recovered om the subsequent years, with 1.0329 million entries in 2021 and 631,800 entries in 2022 [6]. Despite the challenges posed by the pandemic, China has been actively improving and optimizing its entry and exit policies to facilitate the exchange of people between China and foreign countries. As part of these measures, health assessments have been implemented for individuals seeking long-term visas, such as those for labor services, study abroad, immigration, and other purposes, at Shanghai port. The health examination includes screening for sexually transmitted diseases (STDs), with syphilis being one of the items checked. By implementing the screening program for syphilis, imported cases of the disease can be promptly identified at the port, allowing for follow-up supervision and reducing the risk of syphilis being imported into China. The primary objective of this study is to analyze the prevalence and epidemiological characteristics of syphilis in the entry-exit population at Shanghai port from 2014 to 2022. The findings of this analysis will serve as a basis for developing effective prevention and control measures for syphilis at Shanghai port, aiding in safeguarding public health and preventing the spread of the disease.

## Materials and methods

### Data source

In this study, a total of 593,482 entry-exit individuals who passed through Shanghai port from 2014 to 2022 were selected as the research objects. These individuals included foreign nationals, as well as residents of Hong Kong, Macao, Taiwan, and Chinese nationals holding overseas green cards. All participants were required to undergo an entry-exit physical examination as part of the standard procedure within the information management system of Shanghai International Travel Health Care Center. Data on infectious diseases, particularly cases of syphilis, were collect ed from the records of reported cases within the “Shanghai International Travel Health Center” system network.

### Data quality control

Serological testing for syphilis was conducted annually for all entry-exit populations at Shanghai port, following the Diagnostic Criteria of Syphilis WS273-2018 [7]. To validate the reliability of the syphilis detection process, the researchers cross-checked the data from the information system, including physical examinations and serological testing. As a result, the false positive rate of syphilis detection was found to be less than 0.2%, indicating that the likelihood of incorrectly identifying individuals as having syphilis when they did not have the disease was extremely low. Additionally, the study reported a false positive rate of 0, meaning there were no cases where individuals were mistakenly identified as having syphilis. These findings indicate that the statistical data obtained from the study are representative and relatively reliable, with a high level of accuracy in reflecting the actual detection situation and characteristics of syphilis at Shanghai port.

### Statistical analysis

A database was created using Excel 2007, and SPSS 19.0 was used to analyze the data. The percentage of counting data was calculated, and the trend of change between groups was tested by the trend χ^2^ test, with α = 0.05.

## Results

### Incidence trend

**Fig. 1:**
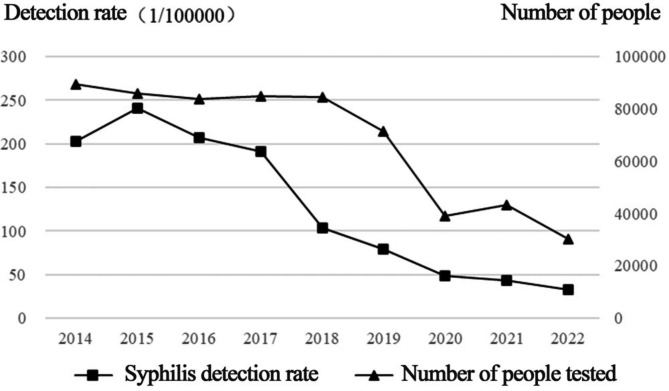
Changes in the detection rate of syphilis from 2014 to 2022 among entry exit population at Shanghai Port, China

From 2014 to 2022, the total number of people who underwent physical examinations at Shanghai port was 593,482, of which 918 had syphilis, and the overall syphilis detection rate was 154.68/100,000. From 2014 to 2022, the number of people who underwent physical examination was 89,641, 85,971, 83,780, 84,891, 84,460, 71,541, 39,113, 29,073, and 25,012, and the syphilis detection rates were 203.03/100 000, 240.78/100 000, 207.69/100 000, 190.83/100 000, 104.19/100 000, 79.67/100 000, 48.58/100 000, 65.35/100 000 and 39.98/100 000, respectively, with the highest rate in 2015 and the lowest rate in 2022. As shown in Table 1; Fig. 1, the syphilis detection rate showed a downward trend and decreased more significantly from 2015 to 2022.

**Table 1:** Changes in regional distribution of syphilis among entry exit population at Shanghai Port, China from 2014 to 2022

Year	North America (n)	Oceania (n)	Southeast Asia (n)	East Asia (n)	Africa (n)	Latin America (n)	Europe (n)	Rest of Asia (n)	Overall (n)
2014	17	6	11	115	2	4	18	9	182
2015	19	3	23	116	10	6	27	3	207
2016	20	3	19	93	2	1	28	8	174
2017	7	4	9	96	8	2	31	5	162
2018	5	2	11	50	3	1	13	3	88
2019	6	2	7	20	3	3	13	3	57
2020	7	0	1	4	1	2	4	0	19
2021	7	0	2	4	2	4	0	0	19
2022	0	3	2	1	1	1	2	0	10
Overall (n)	88	23	85	499	32	24	136	31	918

### Regional distribution

As shown in Table 1, it can be seen that between 2014 and 2022, cases were reported in 8 regions worldwide. Among them, the most syphilis cases were detected in East Asia, with a total of 499 cases, accounting for 54.36%, followed by Europe and North America, accounting for a combined 24.40%. However, the overall downward trend was still relatively obvious.

### Population distribution

Between 2014 and 2022, the male-to-female ratio was 3.91:1 (731:187) (Table 2). The detection rate was higher for males than females, and the overall syphilis detection rate was 202.34/100 000 in males and 74.34/100 000 in females. Most people were aged 30–39 years and 40–49 years, with detection rates of 26.4% and 25.3%, respectively. The detection rate of syphilis in people over 60 years old showed a downward trend from 2014 to 2018 (Table 3).

**Table 2:** Changes in gender distribution of syphilis detection (n[%]) among entry exit population at Shanghai Port, China from 2014 to 2022

Year	Males	Females
2014	137 (75.3)	45 (24.7)
2015	166 (80.2)	41 (19.8)
2016	141 (81)	33 (19)
2017	135 (83.3)	27 (16.7)
2018	69 (78.4)	19 (21.6)
2019	41 (71.9)	16 (28.1)
2020	17 (89.5)	2 (10.5)
2021	17 (89.5)	2 (10.5)
2022	8 (80.0)	2 (20.0)
Overall	731 (79.6)	187 (20.4)

**Table 3:** Changes in age distribution of syphilis detection (n[%]) among entry exit population at Shanghai Port, China from 2014 to 2022

Year	< 29 years	30–39 years	40–49 years	50–59 years	≥ 60 years
2014	3 (1.6)	36 (19.8)	45 (24.7)	51 (28)	47 (25.8)
2015	7 (3.4)	39 (18.8)	64 (30.9)	52 (25.1)	45 (21.7)
2016	14 (8)	44 (25.3)	44 (25.3)	45 (25.9)	27 (15.5)
2017	17 (10.5)	58 (35.8)	39 (24.1)	26 (16)	22 (13.6)
2018	21 (23.9)	26 (29.5)	18 (20.5)	17 (19.3)	6 (6.8)
2019	15 (26.3)	19 (33.3)	14 (24.6)	5 (8.8)	4 (7)
2020	3 (15.8)	9 (47.4)	5 (26.3)	2 (10.5)	0 (0)
2021	8 (42.1)	6 (31.6)	1 (5.3)	4 (21.1)	0 (0)
2022	1 (10.0)	5 (50.0)	2 (20.0)	1 (10.0)	1 (10.0)
Overall	89 (9.7)	242 (26.4)	232 (25.3)	203 (22.1)	152 (16.6)

#### Gender distribution

As shown in Table 2, among people with positive syphilis test results, the proportion of males was higher than that of females (79.63% vs. 20.37%). The detection rate was higher in males than females, with an overall detection rate of 202.34/100,000 in males and 74.34/100,000 in females, respectively. The chi-square test showed that the sex distribution of syphilis did not change with the change of year (χ2 = 1.009, P > 0.05).

#### Age distribution

As shown in Table 3, there were 89 (9.7%), 242 (26.4%), 232 (25.3%), 203 (22.1%), and 152 (16.6%) people aged < 29 years, 30–39 years, 40–49 years, 40–49 years and ≥ 60 years, respectively, with the highest proportion of individuals aged 30–39 years. The chi-square test for trend results showed that the distribution of age at syphilis detection changed as the years changed (χ^2^ = 100.07, P < 0.05).

### Distribution of changes in infection routes

Table 4 shows that from 2014 to 2022, the transmission route of syphilis was mainly sexual transmission, with a total of 559 cases, accounting for 60.9% of the total detected persons. Among them, 125 cases were infected by MSM, accounting for 22.36% of the persons infected by sexual transmission. With the increase in years, the transmission route of syphilis-detected persons did not change significantly (χ^2^ = 0.170, P > 0.05).

**Table 4:** Changes in the distribution of transmission routes of syphilis detection (n [%]) among entry exit population at Shanghai Port, China from 2014 to 2022

	Sexual transmission			Mother-to-fetus transmission	Unclear
Year	Total	Male-to-male transmission	Blood-borne transmission		
2014	121 (66.5)	17 (9.3)	4 (2.2)	0 (0)	57 (31.3)
2015	115 (55.6)	24 (11.6)	5 (2.4)	0 (0)	87 (42)
2016	110 (63.2)	24 (13.8)	6 (3.4)	0 (0)	58 (33.3)
2017	98 (60.5)	26 (16)	2 (1.2)	2 (1.2)	60 (37)
2018	47 (53.4)	14 (15.9)	0 (0)	0 (0)	41 (46.6)
2019	35 (61.4)	15 (26.3)	1 (1.8)	1 (1.8)	20 (35.1)
2020	14 (73.7)	2 (10.5)	0 (0)	0 (0)	5 (26.3)
2021	12 (63.2)	2 (10.5)	0 (0)	0 (0)	7 (36.8)
2022	7 (70.0)	1 (10.0)	0 (0)	0 (0)	3 (30.0)
Overall	559 (60.9)	125 (13.6)	18 (2.0)	3 (0.3)	338 (36.8)

## Discussion

Imported infectious diseases have become a significant concern in China’s efforts to prevent and control infectious diseases, as they are closely linked to the overall health and well-being of the nation’s economy and society. The results of syphilis surveillance among entry-exit personnel at Shanghai port from 2014 to 2022 revealed that sexual transmission was the primary route of syphilis transmission, and the prevalence of syphilis varied significantly among different regions. The high detection rate of syphilis among entry-exit personnel from East Asia, Europe, and North America was attributed to several factors, including the relatively open sexual attitudes in those countries, the low attention to infectious diseases, and high-risk sexual behaviors. To prevent the cross-border spread of infectious diseases, it is crucial for the port authorities to focus on foreigners in their infectious disease surveillance efforts, particularly those coming for business, residence, or study abroad. Greater attention should be paid to the prevention and control of syphilis among this population. Among the different regions, East Asia had the highest syphilis detection rate, but from 2014 to 2022, there was a declining trend in the detection rate year by year. This decline suggests that efforts to combat syphilis in the region have been relatively effective during the study period. In terms of gender, the detection rate of syphilis was higher in males than in females. This difference may be attributed to males’ more extensive social activities, wider social circle, and increased likelihood of exposure to syphilis and other pathogens, leading to a higher risk of infectious diseases. Additionally, it could be related relatively weaker awareness among males abut protection against infectious diseases. Regarding age characteristics, the 30–39 age group had a higher detection rate of syphilis compared to other age groups, while the detection rate was low among middle-aged and older individuals over 60 years old. Interestingly, the detection rate of syphilis in the age group under 29 years old showed a significant increase, indicating a need for enhanced education targeting young students. Timey detection and management of the disease among individuals with clinical manifestations, are crucial, and preventive measures should be promptly implemented to reduce the prevalence of syphilis in campus settings.

Syphilis is one of the global public health problems. In recent years, the number of syphilis cases in the world has decreased significantly [1]. Firstly, with the popularization of health education, people’s health concepts and awareness of seeking medical treatment have been gradually improved. Second, with the development of the science and technology economy, global social health security has been valued, and the means of monitoring and the level of technology and funding support are continuously increasing. Early diagnosis and treatment are essential to reduce the prevalence of syphilis. At present, serological detection of syphilis is still the gold standard for diagnosis, and PCR nucleic acid amplification to detect Treponema pallidum DNA may be a more promising method [8]. Affected by the COVID-19 epidemic, the number of inbound arrivals in China has decreased significantly. Some studies have analyzed the changes in syphilis cases reported before and during the COVID-19 epidemic in China [9]. In 2020 and 2021, the reported syphilis cases in China decreased by 13.32% and 10.41% compared with those before the epidemic, respectively. This study suggests that even if the number of reported cases of syphilis decreases significantly, the decline in the incidence of syphilis should not be considered a future trend, and it is necessary to scientifically re-evaluate the changes in the prevalence of syphilis. Some findings have shown that the incidence of syphilis is positively correlated with the gross domestic product (GDP) per capita, the number of health technicians per 10,000, the proportion of the elderly, and the temperature, and negatively correlated with the proportion of the urban population, the proportion of males, and the precipitation, and those areas at high risk of syphilis are mainly located in more developed coastal areas where more targeted interventions need to be implemented [10]. After the COVID-19 epidemic, with the further expansion of opening up to the outside world, the number of syphilis cases in Shanghai, as an important exit and entry barrier, is expected to show an upward trend. Understanding the epidemiological characteristics of syphilis at Shanghai port is of great significance for the surveillance, prevention, and control of syphilis at Shanghai port.

East Asia, encompassing countries like China, Mongolia, North Korea, South Korea, and Japan, is one of the most densely populated regions in the world. According to a report from the WHO, when compared to Africa and other regions, the proportion of syphilis infection in East Asia is relatively low [11]. However, there are specific risk factors that contribute to a higher prevalence of syphilis among travelers at Shanghai port, such as chronic diseases, unhealthy lifestyle habits, and lower immunity. This highlights the importance of vigilance in monitoring and addressing syphilis cases in this region, particularly among travelers from Southeast Asia, North America, and other areas. A global pooled estimate for syphilis prevalence in MSM between 2000 and 2020 was reported to be 7.5% (95% CI 7.0–8.0, 345 data points; n = 606,232). Three of the eight Sustainable Development Goal (SDG) regions, namely Latin America and the Caribbean, Northern Africa and Western Asia, and Eastern and South-Eastern Asia, had pooled prevalence estimates higher than the global Fig. [2].

With the gradual opening of people’s sexual concepts, the application of social networks, and the increase of anonymity in human contact, sexual partner management has become more challenging [12], leading to an increase in the incidence of unsafe sexual behaviors such as commercial sex and sex between males [13]. In 2018, 86% of syphilis patients in the United States were males, and more than half of those with syphilis reported having sex with males [14]. In Europe, there has been a similar increase in syphilis among MSM [4]. At the same time, the rate of serological screening for syphilis in high-risk groups is relatively lagging behind, and there is a lack of effective syphilis vaccine [15, 16]. The prevalence of syphilis remained high among MSM and male clients in STD clinics. It is necessary to conduct health education and promote condom use among MSM to reduce the spread of syphilis. The results of a cross-sectional study of sexually transmitted infections among MSM in China showed that the use of dating apps among MSM was higher, which might be related to the higher incidence of syphilis [17].

This study provides valuable insights into the results of syphilis screening among entry personnel at Shanghai port from 2014 to 2022. This analysis is instrumental in managing imported syphilis cases among entry and exit personnel and in preventing the spread of syphilis through border ports. As economic globalization continues to progress, China is facing an increasing number of imported syphilis patients, posing challenges to port health quarantine and requiring the implementation of new technologies and supervision methods. In response to these challenges, the customs in the Yangtze River Delta have established a coordinated disposal mechanism for major imported infectious diseases. This mechanism facilitates the sharing of real-time surveillance data and innovative management approaches for patients with imported infectious diseases. Despite efforts, the epidemic situation of syphilis remains serious in the floating population residing in China’s border areas. Therefore, it is crucial to strengthen syphilis surveillance in these regions. Given that syphilis infection is closely related to exposure factors and population characteristics, enhancing surveillance in high-risk areas and high-risk groups, as well as regularly reexamining confirmed cases, is imperative. Additionally, reviewing medium and short-term visitors meticulously is essential to avoid overlooking potential cases during port inspections. To effectively curb the continuous importation of syphilis, further improvements to relevant laws and regulations are necessary. As the world’s largest port city, Shanghai’s epidemiological investigation and prevention measures against syphilis hold significant reference value for both domestic and international syphilis surveillance efforts. It is vital to explore and establish a more efficient and accurate syphilis supervision model to ensure public safety effectively.

## Conclusions

This study has shown that the detection rate of syphilis among entry-exit personnel at Shanghai port has been decreasing continuously in recent years. Geographically, East Asia had the most reported cases, followed by Europe and North America. In terms of population distribution, males had a higher syphilis detection rate than females, with the most affected age groups being 30–39 and 40–49 years. Sexual transmission remained the predominant route of infection, accounting for the majority of cases, with no significant change over the years. Targeted health intervention should be carried out according to the monitoring results.

## Data Availability

The data will be available on request to the corresponding author.

## References

[1] Chlamydia. gonorrhoea, trichomoniasis and syphilis: global prevalence and incidence estimates, 2016. 10.2471/BLT.18.228486 PMC6653813 31384073

[2] Motoyuki Tsuboi J, Evans EP, Davies J, Rowley EL, Korenromp T, Clayton MM, Taylor D, Mabey. R Matthew Chico. Prevalence of syphilis among men who have sex with men: a global systematic review and meta-analysis from 2000-20. Lancet Global Health. 10.1016/S2214-109X(21)00221-7 PMC9150735 34246332

[3] World Health Organization. Report on globally sexually transmitted infection surveillance,2015. http://apps.who.int/iris/bitstream/handle/10665/249553/9789241565301-eng.pdf;jsessionid=25DA348EE7CDFC387C271F7767AFD756?sequence=1a (Accessed on May 16, 2018).

[4] Spiteri G, Unemo M, Mårdh O, Amato- Gauci AJ. The resurgence of syphilis in high-income countries in the 2000s: a focus on Europe[J]. Epidemiol Infect 2019, 147e143. 10.1017/S0950268819000281 PMC6518487 30869043

[5] Tao Y, Chen MY, Tucker JD (2020). A nationwide spatiotemporal analysis of syphilis over 21 years and implications for prevention and control in China[J]. Clin In- fect Dis.

[6] Shanghai Statistics Bureau. http://tjj.sh.gov.cn/ydshj/index.html.

[7] People’s. Republic of China health industry standard. Diagnosis for syphilis. http://www.fjcdc.com.cn/upload/content/file/2019/20190506151253_9750.pdf.

[8] Luo Y, Xie Y, Xiao Y (2021). Laboratory Diagnostic Tools for Syphilis: current status and future prospects. Front Cell Infect Microbiol.

[9] Wu YL, Zhu WQ, Yue XL, Li J, Zhang JH, Gong XD (2022). Zhonghua Liu Xing Bing Xue Za Zhi.

[10] Zhu X, Zhu Z, Gu L (2022). Spatio-temporal variation on syphilis from 2005 to 2018 in Zhejiang Province, China. Front Public Health.

[11] Global progress report on HIV, viral hepatitis and sexually transmitted infections, 2021 (who.int).

[12] de Voux A, Bernstein KT, Bradley H, Kirkcaldy RD, Tie Y, Shouse RL (2019). Syphilis testing among sexually active men who have sex with men and who are receiving medical care for human immunodeficiency virus in the United States: medical monitoring Project[G]. Clin In- fect Dis.

[13] Kidd SE, Grey JA, Torrone EA, Wein stock HS (2019). Increased methamphetamine, injection drug, and heroin use among women and heterosexual men with primary and secondary syphilis United States[G]. MMWR Morb Mortal Wkly Rep.

[14] Spicknall IH, Kreisel KM, Weinstock HS (2021). Estimates of the prevalence and incidence of Syphilis in the United States, 2018. Sex Transm Dis.

[15] Traeger MW, Cornelisse VJ, Asselin J (2019). Association of HIV preexposure pro- phylaxis with incidence of sexually trans- mitted infections among individuals at high risk of HIV infection[J]. JAMA.

[16] Molina JM, Charreau I, Chidiac C (2018). Post-exposure prophylaxis with doxy- cycline to prevent sexually transmitted infections in men who have sex with men: an open-label randomisedsubstudy of the ANRS IPERGAY trial[J]. Lancet Infect Dis.

[17] Guo Z, Feng A, Zhou Y (2023). Geosocial networking mobile applications use and HIV and other sexually transmitted infections among men who have sex with men in Southern China: a cross-sectional study. Front Public Health.

